# Qualitative Changes in Cortical Thymic Epithelial Cells Drive Postpartum Thymic Regeneration

**DOI:** 10.3389/fimmu.2019.03118

**Published:** 2020-01-15

**Authors:** Maude Dumont-Lagacé, Tariq Daouda, Lucyle Depoërs, Jérémie Zumer, Yahya Benslimane, Sylvie Brochu, Lea Harrington, Sébastien Lemieux, Claude Perreault

**Affiliations:** ^1^Immunobiology Research Unit, Department of Medicine, Institute for Research in Immunology and Cancer, Université de Montréal, Montréal, QC, Canada; ^2^Functional and Structural Bioinformatics Research Unit, Department of Biochemistry and Molecular Medicine, Institute for Research in Immunology and Cancer, Université de Montréal, Montréal, QC, Canada; ^3^Telomere Length Homeostasis and Genomic Instability Research Unit, Department of Medicine, Institute for Research in Immunology and Cancer, Université de Montréal, Montréal, QC, Canada

**Keywords:** thymic regeneration, thymic epithelial cells, postpartum thymic regeneration, RNA sequencing, thymopoiesis

## Abstract

During gestation, sex hormones cause a significant thymic involution which enhances fertility. This thymic involution is rapidly corrected following parturition. As thymic epithelial cells (TECs) are responsible for the regulation of thymopoiesis, we analyzed the sequential phenotypic and transcriptomic changes in TECs during the postpartum period in order to identify mechanisms triggering postpartum thymic regeneration. In particular, we performed flow cytometry analyses and deep RNA-sequencing on purified TEC subsets at several time points before and after parturition. We report that pregnancy-induced involution is not caused by loss of TECs since their number does not change during or after pregnancy. However, during pregnancy, we observed a significant depletion of all thymocyte subsets downstream of the double-negative 1 (DN1) differentiation stage. Variations in thymocyte numbers correlated with conspicuous changes in the transcriptome of cortical TECs (cTECs). The transcriptomic changes affected predominantly cTEC expression of *Foxn1*, its targets and several genes that are essential for thymopoiesis. By contrast, medullary TECs (mTECs) showed very little transcriptomic changes in the early postpartum regenerative phase, but seemed to respond to the expansion of single-positive (SP) thymocytes in the late phase of regeneration. Together, these results show that postpartum thymic regeneration is orchestrated by variations in expression of a well-defined subset of cTEC genes, that occur very early after parturition.

## Introduction

Thymic involution that occurs during pregnancy is characterized by an important decrease in thymic mass and cellularity. This decrease affects all thymocyte subpopulations, including CD4^−^CD8^−^ double negative (DNs), CD4^+^CD8^+^ double positive (DP) and single positive (SP) CD4^+^ and CD8^+^ thymocytes (1, 2). This phenomenon of acute thymic involution is not caused by massive apoptosis of DP thymocytes, as found following sublethal irradiation or dexamethasone administration, but rather through a pause in thymocyte differentiation and proliferation (1). That blockade in thymocyte development occurs between the DN1 and DN2 stages (CD44^+^CD25^−^ and CD44^+^CD25^+^, respectively), leading to an accumulation of DN1 thymocytes during gestation (3). Pregnancy-induced thymic involution is conserved in all mammalian species examined and certainly represents a major biological challenge. Indeed, failure to regain immunocompetence would jeopardize survival of both females and their progeny (4).

Pregnancy-associated thymic involution is dependent on the presence of sex hormones produced during gestation, i.e., estrogens and progesterone (5). However, sex hormones do not act directly on thymocytes, but rather through stromal mediators. Indeed, sex hormones have been shown to have pervasive effects on TECs (6, 7). In particular, the expression of the progesterone receptor (*Pgr*) specifically by the thymic stromal compartment is necessary and sufficient for pregnancy-associated involution to occur (8). This suggests that progesterone induces alterations in the thymic stromal niche responsible for DN thymocytes development. Many genes involved in thymocyte development are downregulated during pregnancy: (i) the chemokines *Ccl25, Ccl21/19, Cxcl12*, which encode for ligands of CCR9, CCR7, and CXCR4, respectively, necessary for thymocytes migration in the thymus (3), (ii) the cytokines interleukin-7 (*Il7*) and *Kitl*, essential for thymocytes expansion and maturation (9–11), and (iii) *Dll4*, a Notch ligand essential for lymphoid precursors differentiation toward the T cell lineage (12, 13).

Recent studies have identified important players in adult thymic regeneration using other models of thymic involution. Bredenkamp et al. (14) showed that overexpression of *Foxn1* in TECs, a key transcription factor involved in TEC differentiation and necessary for the expression of genes involved in the regulation of thymopoiesis, was sufficient to induce thymic regeneration of fully involuted thymus in aged mice (12 or 24 months old) (14). Wertheimer et al. (15) more recently showed that production of BMP4 by thymic endothelial cells promoted thymic regeneration following sublethal irradiation, and acted by inducing the expression of *Foxn1* and its targets in TECs (15). However, we do not know whether these mechanisms are at play in other models, such as postpartum thymic regeneration. More specifically, very little is known on the sequence of events and the cells responsible for triggering and regulating thymic regeneration.

In an effort to identify factors involved in adult thymic regeneration, we analyzed TEC and thymocyte subpopulations during the postpartum thymic regenerative phase. In this study, we show that pregnancy-induced thymic involution is not associated with cell loss in the thymic epithelium, and that postpartum thymic regeneration is orchestrated by transcriptomic and phenotypic changes occurring primarily in cortical TECs (cTECs).

## Materials and Methods

### Murine Experiments

All mice were bred and housed under specific-pathogen-free conditions in sterile ventilated racks at the Institute for Research in Immunology and Cancer. B.6SJL-Ptprc^a^ Pep3^b^/BoyJ-CD45.1 female mice (Jackson Laboratory, #002014, also called B6.SJL) were mated with BALB/cJ males (Jackson Laboratory, #000651). Allogeneic mating was selected in order to increase the frequency of successful mating. No differences were observed in the pregnancy-induced thymic involution or the rate of thymic regeneration postpartum in females following syngeneic or allogeneic mating. As lactation causes a delay in postpartum thymic regeneration, pups were removed at birth. Recent thymic emigrants were measured in RAG2p-GFP mouse [FVB-Tg(Rag2-EGFP)1Mnz/J, Jackson Laboratory, #005688], which were kindly provided by Dr. Moutih Rafei. All procedures were in accordance with the Canadian Council on Animal Care guidelines and approved by the Comité de Déontologie et Expérimentation Animale de l'Université de Montréal.

### Enzymatic Digestion of Thymic Stroma

Stromal cell enrichment was performed as described (16, 17) Briefly, thymi were cut in small pieces in RPMI with HEPES (Gibco) at room temperature (r.t.), and gently agitated using P1000 pipet with wide-bore tips to remove thymocytes. This step was repeated until no more thymocytes were released (supernatant stayed translucid). Then, the supernatant was removed and replaced with 1 mL of RPMI with HEPES containing papain 0.5 mg/ml (Worthington Biochemical Corporation), DNase 0.1 mg/ml (Sigma) and collagenase IV 0.25 mg/ml (Sigma) and incubated at 37°C for 3 × 15 min. Stromal fragments were agitated using wide-bore tips first, and then standard P1000 tips between each incubation. After the second incubation, supernatant was removed and placed in a falcon tube containing 5 ml of PBS 1X with 0.5% bovine serum albumin (Sigma) at r.t. and the remaining stromal fragments were incubated once again with 1 ml of enzyme solution. Cells are pooled after the third incubation, centrifuged at 4°C and filtered. At this point, cells were kept at 4°C (or on ice) for antibody staining and analysis. Analysis of thymic epithelial populations were performed following enrichment of EpCAM^+^ cells using anti-EpCAM microbeads (mouse, Miltenyi) and LS or MS columns (Miltenyi Biotec), depending on the total number of cells after enzymatic digestion. The list of antibodies used for flow cytometry analyses can be found in Supplementary Table 1. Throughout the paper, TECs are defined as EpCAM^+^CD45^−^, while the cTEC and mTEC subsets were defined as UEA1– and UEA1+ TECs, respectively (see Supplementary Figure 1 for gating strategy).

### RNA-Sequencing

Poly-A enriched mRNA sequencing was performed on cTECs and mTECs. Purified cell populations from non-pregnant females (NP), pregnant females at 18 days of gestation (D-2) and following parturition (D0–D28) were extracted using fluorescence activated cell sorter (FACS) after enzymatic digestion of the stroma. Each replicate was extracted from one mouse (each sample containing between 13,800 and 96,700 cells). RNA extraction was performed using Trizol™ as recommended by the manufacturer (Invitrogen), and purified using RNeasy Micro columns (Qiagen). Samples quality was confirmed using Bioanalyzer RNA Pico (Agilent). Transcriptome librairies were synthesized with KAPA RNA HyperPrep PolyA (Roche), and validated with Bioanalyzer (Agilent). Sequencing was performed with the Nextseq500 (NextSeq High Output, 75 cycles) at the genomic core facility of the Institute for Research in Immunology and Cancer (Université de Montréal).

Adapter sequences and 3′ regions of low quality were removed using Trimmomatic (version 0.35) (18). Reads were aligned to mouse reference genome (GRCm38) with STAR version 2.5.1b (19) and gene expression was quantified with RSEM tool and expressed as Fragments Per Kilobase of transcript per Million mapped reads (FPKM) (20).

Subsequent analyses were performed using the publicly available statistical software package “R” (http://www.r-project.org/). Lowly expressed genes (average expression < 0.5 FPKM) were removed from analyses.

### Identification and Analysis of DEGs

Differentially expressed genes (DEGs) between D-2 and D6 were identified using the limma-voom package (21) in R software with thresholds of FC ≥ 2 and an adjusted *p* ≤ 0.01. Gene ontology (GO) enrichment analysis was performed using the DAVID database (https://david.ncifcrf.gov/) (22, 23).

## Results

### Thymic Epithelium Does Not Suffer Cell Loss During Pregnancy

**Figure 1:**
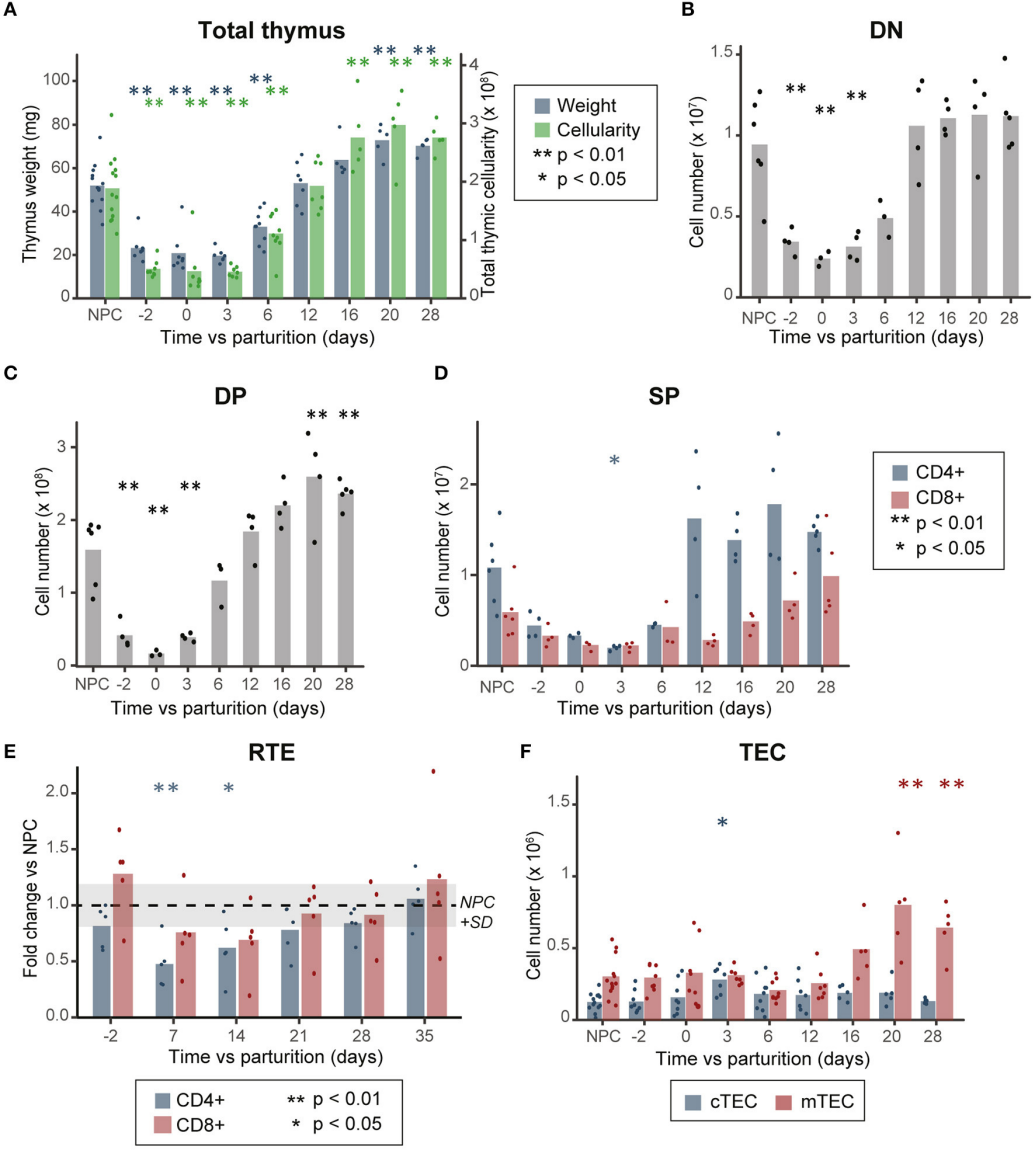
Thymocyte loss during pregnancy does not correlate with a decrease in TEC numbers. **(A)** Thymic mass (mg) and cellularity (×10^8^, *n* = 5–13). **(B)** Cell numbers of DN, **(C)** DP, and **(D)** SP thymocytes (*n* = 3–6). **(E)** Variation in thymic output, as measured by the percentage of recent thymic emigrants (RTE, *n* = 5). **(F)** TEC numbers (×10^6^, *n* = 5–13). NPC refers to the non-pregnant controls, D-2 to pregnant females at day 18 of gestation and D0 to the day of parturition. All analyses were performed in thymi of B6.SJL mice except for those depicted in **(E)** where we used peripheral blood of RAG2p-GFP female mice. Means are displayed as bars, each dot representing an individual. Significance was assessed with one-way ANOVA followed by *post-hoc* Tukey test. *p*-values (*p < 0.05 and **p < 0.01) are shown against NPC only.

We confirmed that during pregnancy, the thymus undergoes a transient involution characterized by a ~60 and ~80% decrease in thymic mass and cellularity, respectively, compared to non-pregnant controls (NPC, Figure 1A) (1, 3). This involution is caused by a depletion of all thymocyte subsets (Figures 1B–D), due to an arrest in thymocyte proliferation and differentiation at the DN1–DN2 stage. This translates into an increase of DN1 proportions in the thymus (Supplementary Figure 2A) (1, 3).

To understand the factors involved in postpartum thymic regeneration, we studied lymphocyte and epithelial cell populations in the first month following parturition. As lactation prevents rapid thymic rebound following parturition (2, 24), pups were removed at birth. The blockade of DN1–DN2 thymocyte differentiation resolved shortly after parturition between day 3 (D3) and D6 postpartum, after which the proportion of DN1 cells returned to age-matched levels (Supplementary Figure 2A). Global thymic cellularity and the size of each thymocyte subset reached age-matched levels within 12 days postpartum (Figures 1A–D). Thereafter, the thymus reaches a hypertrophic state between D16 and D28, when its mass and cellularity reached 1.3–1.5× that of NPC (Figure 1A), mostly due to the accumulation of DP thymocytes (Figure 1C). Interestingly, CD4^+^ and CD8^+^ SP thymocytes (SP4 and SP8, respectively) showed slightly different patterns of reconstitution during pregnancy-induced involution and postpartum regeneration (Figure 1D). While both levels of SP4 and SP8 decrease around parturition and rebound between D12 and D28, the reduction in SP4 cell loss was more pronounced and its rebound more rapid than that of SP8 thymocytes. Accordingly, the decreased proportion of recent thymic emigrants (GFP+ cells) in peripheral blood of RAG2p-GFP mice reached statistical significance (*p* < 0.05) at D7 and D14 postpartum for CD4 T cells, but not for CD8 T cells (Figure 1E).

As all thymocyte populations undergo significant cell loss during gestation, we sought to determine if the thymic epithelium also suffered cell loss during this process. Contrary with previous observations (3), we did not observe any decrease in TEC numbers during gestation or in the postpartum period (Figure 1F and Supplementary Figure 1). This difference could be caused by the use of different enzymatic digestion protocols in the two studies: while the first study used a mix of Dispase I, collagenase II and DNase1, we used a mix of papain, DNase1 and collagenase IV, optimized for maximal recovery of live TECs (16). Of note, the papain-based digestion method that we used yielded ~5× more TECs per thymus, with an average of 7.5 × 10^4^ TECs when using the Dispase I enzyme mix (3) compared to 4 × 10^5^ TECs when using the papain mix (Figure 1C). We conclude that pregnancy-induced thymic involution is not caused by depletion of TECs.

While cTEC numbers increased transiently at D3 (on average ~2.5× higher compared to NPC), prior to the expansion of thymocyte subpopulations, their number returned to NPC levels at D6 and remained stable during the whole process of thymocyte regeneration (Figure 1F). As cTEC and mTEC numbers are similar to NPC during the active thymic growth phase (D6 and D12, Figure 1F and Supplementary Figure 2B), this suggests that no increase in TEC numbers is necessary for postpartum regeneration. Nonetheless, mTEC numbers increased during the late phase of postpartum thymic regeneration (1.6–2.6-fold vs. NPC between D16 and D28, Figure 1F) in synchrony with the apex of thymocyte rebound (Figure 1A). This suggests that the expansion of SP thymocytes occurring between D12 and D28 might have led to the increase in mTEC numbers. This is consistent with the fact that the development of the mTEC compartment during embryogenesis requires interaction between TECs and positively selected thymocytes (25–31), and suggests that a similar crosstalk is instrumental to the maintenance of mTECs in the adult thymus. Representative profiles of cTECs and mTECs at different time points during pregnancy and postpartum regeneration are shown in Supplementary Figure 1.

### Expression of *Foxn1* Is Modulated During Postpartum Regeneration

As the swift postpartum thymic regrowth does not require TEC expansion, we hypothesized that it might rather depend on qualitative changes in TECs. We therefore performed RNA-sequencing of cTECs and mTECs extracted from NPCs, and at 15 time points spanning between D-2 (i.e., 2 days before parturition) to D28 postpartum (Supplementary Figure 3). Based on the analysis of cell population numbers (Figure 1), we distinguished two phases in the process of postpartum regeneration: (i) D0–D12: early regeneration, during which thymic cellularity gradually increases, and (ii) D16–D28: late regeneration, when thymic cellularity exceeds that of NPC. We first analyzed the two genes previously reported to be instrumental in other models of thymic regeneration in adults: *Foxn1* and *Bmp4* (14, 15).

**Figure 2:**
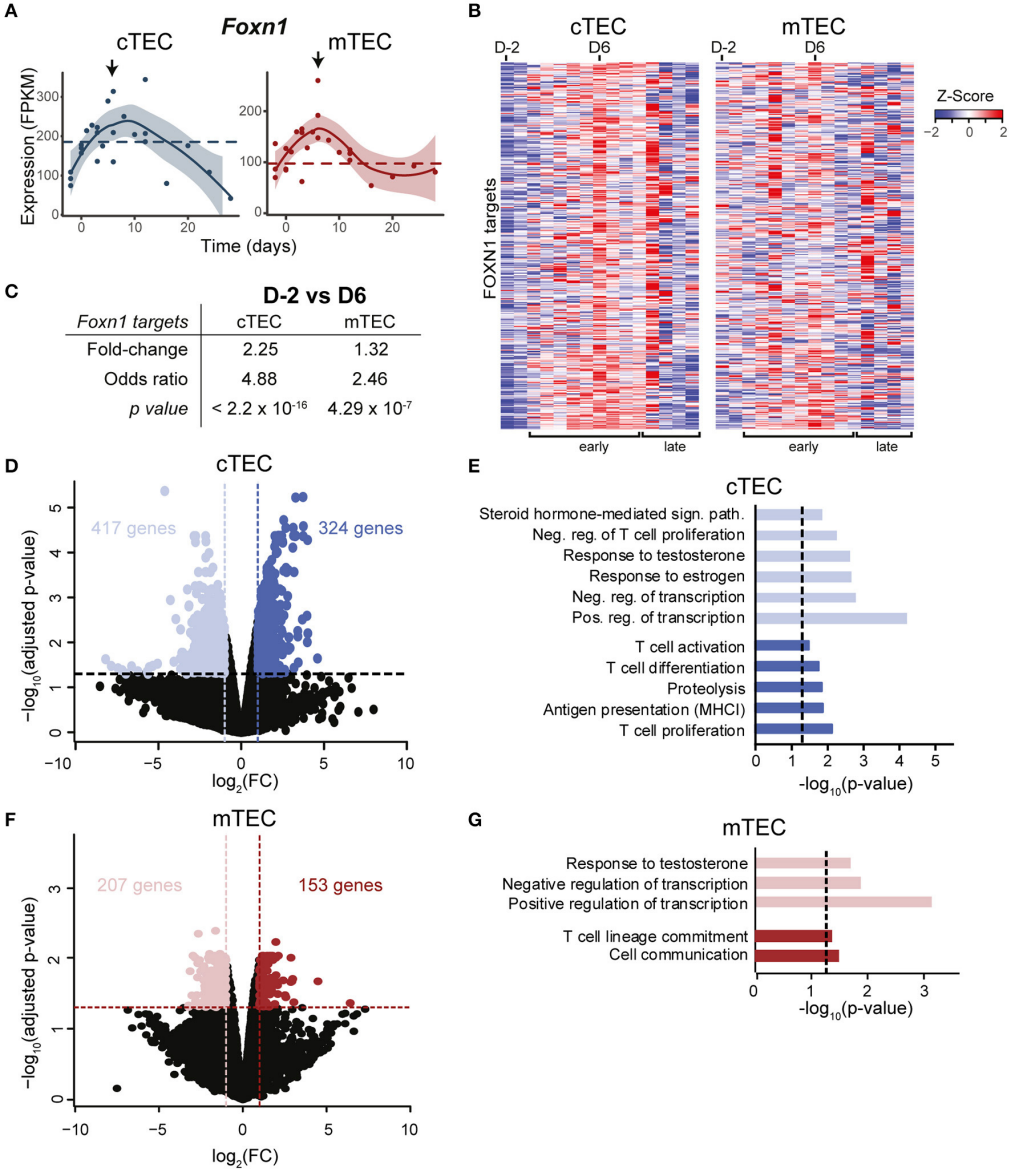
Transcriptomic changes in TECs during postpartum regeneration. **(A)** *Foxn1* expression in cTECs and mTECs. Each replicate is represented with a dot. Black lines represent non-linear regression and the 95% confidence intervals are displayed as shaded areas (blue for cTECs, red for mTECs). Average expression in NPC is depicted with a dashed line. **(B)** Expression of FOXN1 target genes, as defined by (33). Gene expression is shown as relative expression (*Z*-score), for D-2 to D28 (one column per time point). **(C)** Average fold-change in expression of FOXN1 targets between D-2 and D6. Odds Ratio and significance were assessed with Fisher exact test, for fold-change > 2. Gene expression for each time point represents either average expression for triplicates, or raw expression value for individual replicates. **(D)** Differential gene expression in cTECs between D-2 (pale blue) and D6 (dark blue). **(E)** Enriched GO-terms in cTECs between D-2 and D6. **(F)** Differential gene expression in mTECs between D-2 (pale red) and D6 (dark red). **(G)** Enriched GO-terms in mTECs between D-2 and D6.

*Foxn1*, a transcription factor expressed by TECs, is essential for their expansion and differentiation. While *Foxn1* is highly expressed in TECs of young individuals, its expression declines with age, which contributes to senescence-related thymic involution (32). Furthermore, overexpression of *Foxn1* in a severely involuted thymus (in 1 or 2 year-old mice) can induce thymic rejuvenation, characterized by an increased thymic cellularity and by phenotypic qualities associated with young thymi (14). We found that at the end of gestation (D-2), *Foxn1* expression was reduced in cTECs (~2×), but not in mTECs (Figure 2A). During the early regenerative phase, *Foxn1* expression rapidly increased in both cTECs and mTECs (as early as D0 for cTECs, Figure 2A). This translated into upregulation of FOXN1 target genes (33) from D2 postpartum and reaching its maximum around D6 in cTECs (Figures 2B,C). As increased expression of *Foxn1* enhances thymic cellularity and output (34), our results provide strong evidence that *Foxn1* is involved in postpartum thymic regeneration. Notably, the overexpression of *Foxn1* and its target genes in cTECs was transient. Indeed, during the late regenerative phase, their expression regressed to levels found at D-2 (Figures 2A,B). As the downregulation of *Foxn1* with age depends on the presence of differentiating DP and SP thymocytes (32), our data suggest that thymocyte expansion during late postpartum thymic regeneration induces an negative feedback loop that inhibits *Foxn1* expression. Of note, *Foxn1* activation was independent of WNT signaling (Supplementary Figure 4).

A recent study showed that secretion of BMP4 by thymic endothelial cells promotes thymic regeneration following acute involution induced by sub-lethal total body irradiation (15). Increased BMP4 signaling was observed in thymic stromal cells 4–7 days following irradiation, and was accompanied by an upregulation of *Foxn1* and its target genes in TECs. While we have not analyzed BMP4 expression in thymic endothelial cells, we observed that despite expression of BMP receptors in both cTECs and mTECs (Supplementary Figure 5A), expression of BMP target genes was unchanged in the early postpartum regeneration (GO:0030510, Supplementary Figures 5B,C). These results provide strong evidence that BMP4 is not involved in postpartum thymic regeneration. In order to get a global picture of genes that may contribute to postpartum thymic regeneration, we next compared the transcriptome of cTECs and mTECs at the time of maximal thymic involution (D-2) vs. the time of maximal growth (D6) (Supplementary Figure 2B). We identified differentially expressed genes (fold-change ≥ 2 and *p* ≤ 0.01) and performed a GO-term enrichment analysis. Several genes overexpressed in cTECs at D-2 are involved in steroid hormone response (Figures 2D,E) and included the nuclear progesterone receptor *Pgr*, whose expression in thymic stromal cell is necessary for pregnancy-induced involution (8). We also observed on D-2 an upregulation of genes that negatively regulate T cell proliferation (Figure 2E). Consistent with the pronounced expansion of DP thymocytes at D6, genes involved in T cell activation, proliferation and differentiation were upregulated in cTECs during the early regenerative phase (D6). Interestingly, we also observed on D6 an upregulation of genes involved in MHC-I antigen presentation and proteolysis in cTECs, such as *Psmb9, Psmb10*, and *Psmb11*. In mTECs, variations in gene expression were more modest than in cTECs: only 2% of expressed genes were differentially expressed in mTECs (360 genes) as opposed to 4.7% in cTECs (741 genes) (Figures 2D,F). While mTECs also presented an upregulation of genes involved in cell communication and T cell lineage commitment at D6 (Figure 2G), no changes in antigenic presentation were detected. We conclude that pregnancy-induced sex hormone variations have a more dramatic effect on cTECs than mTECs.

### The Pivotal Role of cTECs in Postpartum Thymic Regeneration

**Figure 3:**
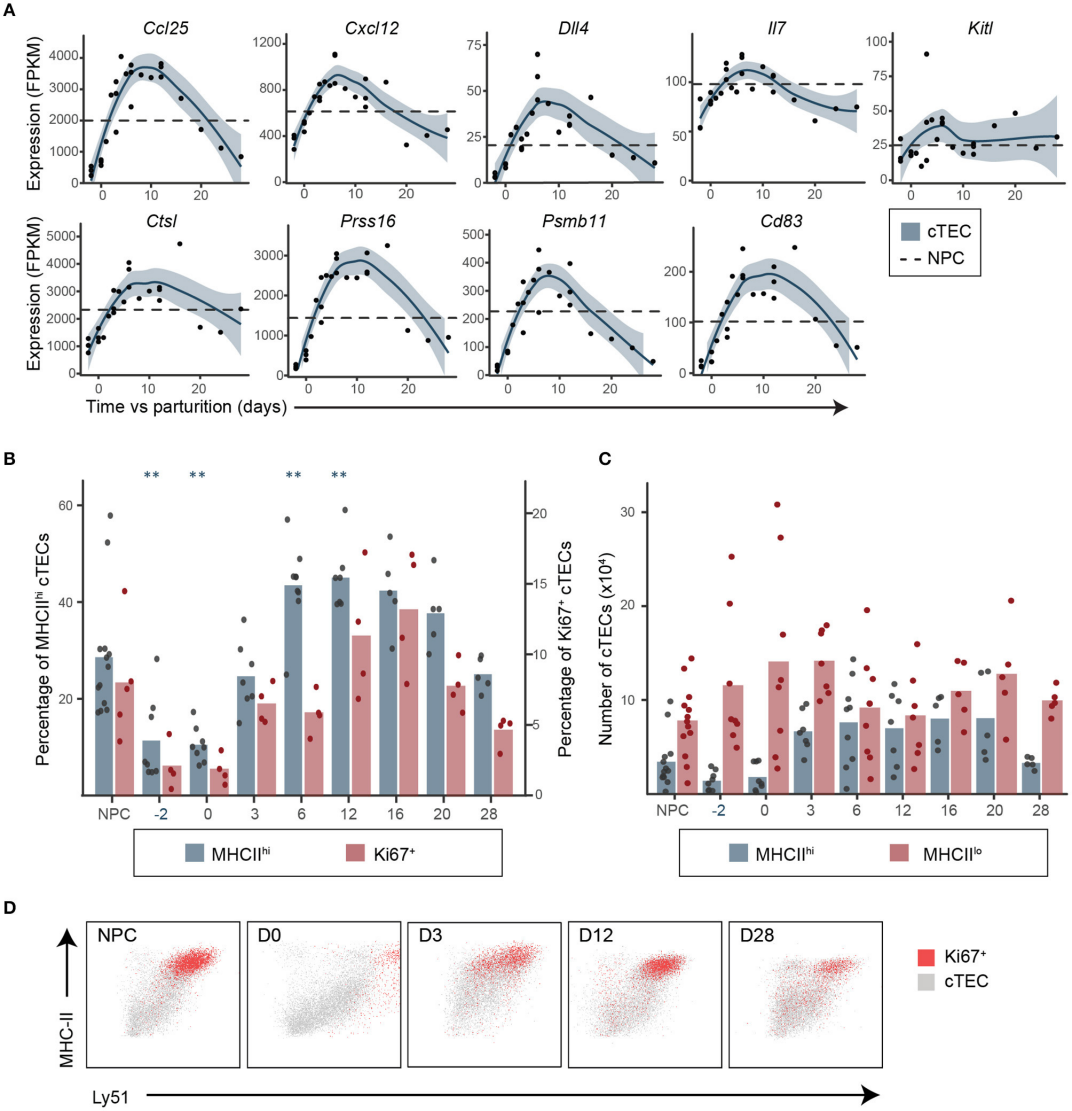
Upregulation of genes involved in thymopoiesis in cTECs precedes the release of thymocyte differentiation blockade in early postpartum regeneration. **(A)** Expression of genes involved in DN thymocytes migration and differentiation and in cTEC antigenic presentation. Each replicate is represented with a dot and a non-linear regression and the 95% confidence intervals (blue shaded area) are displayed. Average expression in NPC is depicted with a dashed line. **(B)** Proportions of MHC-II^hi^ (*n* = 5–13) and Ki67^+^ (*n* = 4–5) cTECs. Means and individual replicates are depicted as bars and dots, respectively. Significance was assessed using one-way ANOVA followed by *post-hoc* Tukey test. *p*-values (**p < 0.01) are shown against NPC only. **(C)** MHC-II^lo^ and MHC-II^hi^ cTEC numbers (×10^6^, *n* = 5–13). **(D)** Representative profiles of MHC-II and Ly51 expression for total (gray) of Ki67^+^ (red) cTECs.

Many FOXN1 targets in cTECs regulate various steps of thymopoiesis: (i) regulation of lymphoid precursors migration in the thymus (*Ccl25* and *Cxcl12*), (ii) DN thymocyte survival and proliferation (*Ccl25, Cxcl12*) and differentiation (*Dll4, Cxcl12*), and (iii) cortico-medullary thymocyte migration (*Psmb11*) (9, 13, 33, 38–44). In cTECs, FOXN1 targets also include genes involved in antigenic presentation essential for positive selection, such as cTEC-specific proteases (*Psmb11, Prss16*, and *Ctsl*) and stimulatory molecules important in TCR signaling (MHC-II, *Cd83*) (45–50). During pregnancy and postpartum regeneration, all these genes followed a pattern of expression similar to that of *Foxn1*: (i) downregulation during pregnancy-induced involution; (ii) upregulation in early regeneration; and (iii) downregulation again in late regeneration (Figure 3A and Supplementary Table 2). Two other cTEC genes play crucial roles in the early stages of thymopoiesis: Il7, which is FOXN1-independent, and Kitl whose FOXN1-dependence is unclear (13, 33, 51). In our mice, the expression of *Il7* followed the pattern of FOXN1-regulated genes, but *Kitl* did not (Figure 3A). Globally, the upregulation of *Il7* and FOXN1-regulated genes (between D0 and D3, Figures 2A,B, 3A,B) preceded the release of DN1–DN2 differentiation (between D3 and D6, Supplementary Figure 2A), supporting the idea that postpartum thymic regeneration was triggered by transcriptomic changes in cTECs.

Upregulation of *Il7* and FOXN1-regulated transcripts coincided with increased proportion and numbers of MHC-II^hi^ cTECs (Figures 3B,C), which include most of the proliferating cTECs (Ki67^+^, Figure 3D). Indeed, the proportion of MHC-II^hi^ cTECs, which was very low at D-2 and during late regeneration, increased during early regeneration when the thymus was in expansion, and decreased at D28 when thymus was hypertrophic (Figures 3C,D). A recent analysis of the thymic stroma in embryonic thymi using single-cell transcriptomics revealed that most of the key cTEC genes modulated during postpartum regeneration (*Foxn1, Ccl25, Cxcl12, Dll4, Ctsl, Prss16, Psmb11, Cd83, Ly51*, and MHC-II) are co-expressed by a single subpopulation of cTECs, which the authors called cTEC4 (52). Therefore, our data suggest that postpartum thymic regeneration may be driven by an expansion of the cTEC4 compartment. It also seems likely that variations in the proportion of MHC-II^hi^ cTECs is strongly associated with the transcriptomic changes observed in cTECs.

### mTECs Proliferate and Differentiate After Thymic Regrowth

**Figure 4:**
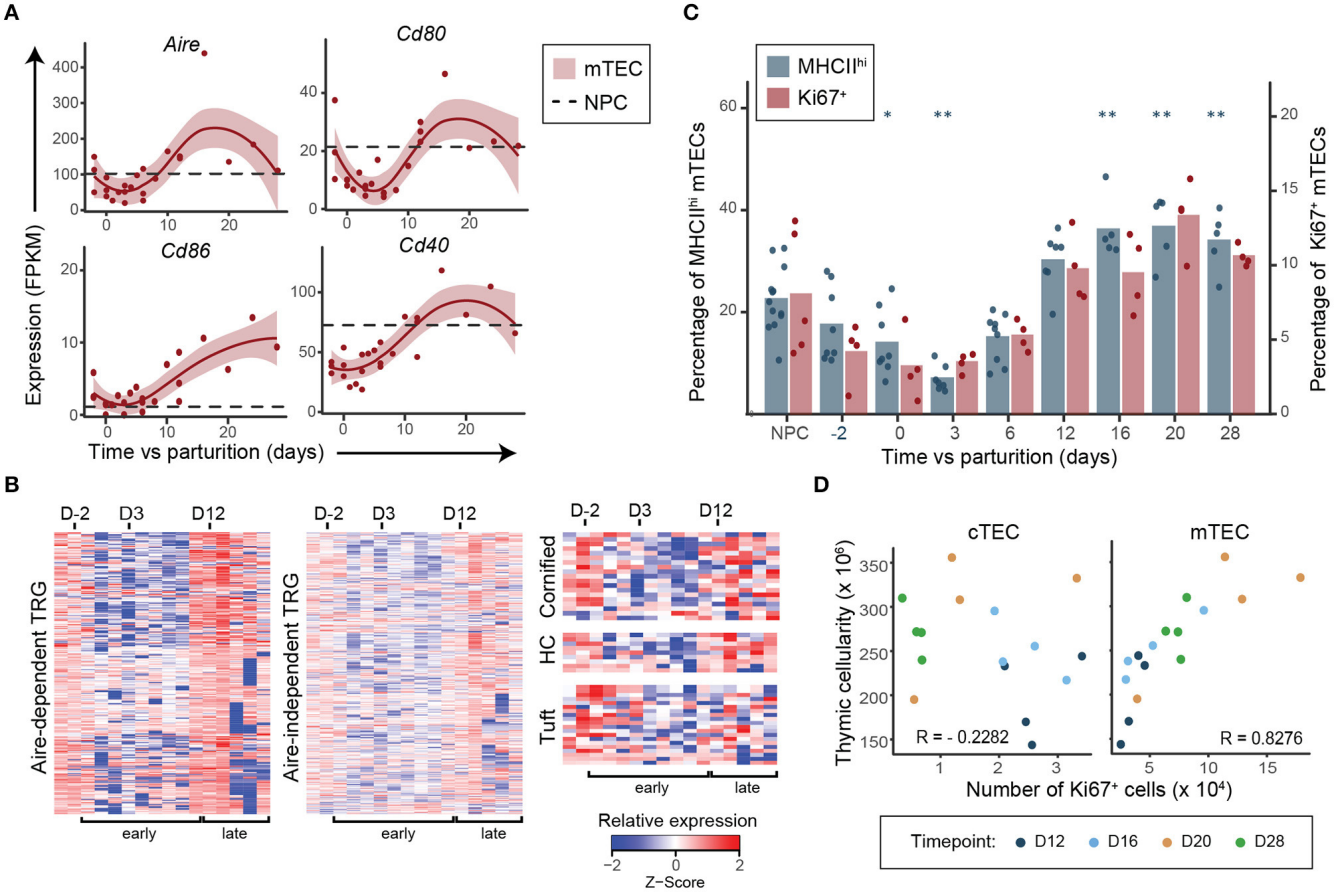
Expansion of SP thymocytes precedes differentiation of mTECs postpartum. **(A)** Expression of genes involved in SP thymocytes negative selection and associated with mTEC maturation. Each replicate is represented with a dot and a non-linear regression and the 95% confidence interval are displayed as lines and shaded area, respectively. Average expression in NPC is depicted with a dashed line. **(B)** Relative expression (*Z*-score) of *Aire*-dependent, *Aire*-independent tissue-restricted genes (TRG) (35), of genes associated with cornified cells, Hassall's corpuscles (HC) and tuft cells (36, 37) in mTECs during postpartum regeneration (NPC is shown in the first left column, then D-2 to D28 postpartum). Gene expression for each time point represents either average expression for triplicates, or raw expression value for single replicate. **(C)** Percentage of MHC-II^hi^ (*n* = 5–13) and Ki67^+^ (*n* = 4–5) mTECs. Means and individual replicate are depicted as bars and dots, respectively. Significance was assessed using one-way ANOVA followed by *post-hoc* Tukey test. *p*-values (*p < 0.05 and **p < 0.01) are shown against NPC only. **(D)** Pearson correlation between global thymic cellularity and the number of Ki67^+^ cTECs or mTECs from D12–D28.

We next analyzed expression of genes associated with mTEC differentiation and involved in SP thymocyte maturation and selection: (i) costimulatory molecules (*Cd80, Cd86*, and *Cd40*), (ii) promiscuously expressed genes (35) and the Autoimmune regulator (*Aire*), and (iii) MHC-II expression at the cell surface (detected by flow cytometry). Interestingly, almost all these molecules (*Cd80, Cd40, Aire*, Aire-dependent and -independent TRGs and MHC-II) were downregulated during early regeneration and upregulated during late regeneration (Figures 4A–C). Genes associated with cornified cells, Hassall's corpuscles and tuft cells (36, 37), which have been associated with different subsets of terminally differentiated mTECs, were also upregulated during the late regenerative phase compared to the early postpartum phase (Figure 4C). These transcriptomic and phenotypical changes in mTECs occurred simultaneously with the increase in mTEC proliferation rate and the increase in proportions of SP thymocytes during the late regenerative phase (Figures 1D, 4C). These results suggest that cross talk with SP thymocytes is required for the maturation of mTECs in the adult thymi, like it is during embryonic development (26). This hypothesis is supported by the correlation between global thymic cellularity and the number of Ki67^+^ mTECs in the late regenerative phase (Figure 4D). Interestingly, no such correlation can be found with the number of Ki67^+^ cTECs (Figure 4D). Taken together, these results suggest that mTECs do not trigger thymic regeneration but rather seem to proliferate and differentiate in response to the increased numbers of SP thymocytes.

## Discussion

We have shown that postpartum thymic regeneration is mostly associated with qualitative changes in the cTEC population, beginning very early after parturition. Contrary to previous results (3), we did not observe a decrease in TEC numbers during pregnancy, the discrepancies most likely explained by the different enzymatic digestion protocols used in the two studies. Nonetheless, both studies have revealed a decrease in the expression of *Ccl25, Cxcl12*, and *Dll4* in cTEC during pregnancy. We have shown that these same genes are also upregulated during early postpartum regeneration, highlighting their importance in the regulation of thymopoiesis. In opposition, mTECs show very little phenotypic changes during gestation: expression of *Foxn1, Cd80, Cd86*, MHC-II, *Aire*, and of tissue-restricted genes are similar at D-2 vs. NPC. These results strongly suggest that the cTECs are the main regulators of the rate of thymopoiesis and are driving both pregnancy-induced involution and postpartum regeneration, while the mTECs proliferate and differentiate in response to the increasing presence of SP thymocytes in the late regenerative phase.

The MHC-II^hi^ subpopulation of cTECs diminishes drastically during gestation, and reappears gradually in early regenerative phase. As this population has been shown to co-express many genes involved in thymopoiesis (such as *Ccl25, Cxcl12, Dll4, Psmb11*, etc.) (52, 53), this suggests that the presence of MHC-II^hi^ cTECs is instrumental in thymic regrowth. Indeed, the majority of cTECs expressed high levels of MHC-II during the most active growth phase of the thymus, from the end of embryonic development (E18) to the postnatal period (54). Interestingly, the MHC-II^hi^ cTEC population also expands in the transient hypertrophy following sex steroids ablation, 7 days after castration (54). Furthermore, the decreased expression of *Foxn1* and its targets in the late regenerative phase, when the number of DP and SP thymocytes was greatly increased, suggests the presence of a negative feedback loop regulating the expression of *Foxn1* in cTECs. This is in line with previous results showing that the presence of more differentiated thymocytes (>DN3) is necessary for the age-related decrease in *Foxn1* expression (32).

In conclusion, our study further extends the pervasive role of *Foxn1* in thymus biology, as it was shown to be involved in all models of thymic growth, regeneration, and involution to this day (14, 15, 34, 53, 55, 56). Also, unlike what is observed following irradiation-induced involution (15), no evidence of BMPR signaling or of damage to the epithelial population was detected in postpartum thymic regeneration. These data imply that the mechanisms involved in triggering thymic regeneration differ depending on the context leading to thymic involution. Moreover, our study demonstrates that the *Foxn1* pathway is tightly regulated in a time sequential manner before and after parturition. This raises a fundamental question: how is *Foxn1* regulated by sex hormones? This regulation could result from direct and/or indirect effects of sex hormones on the *Foxn1* pathway in TECs and is the subject of our ongoing studies.

## Data Availability Statement

The datasets generated for this study can be found in the GEO archives under accession number GSE138494.

## Ethics Statement

The animal study was reviewed and approved by the Comité de Déontologie et Expérimentation Animale de l'Université de Montréal.

## Author Contributions

Experimental design and bioinformatic analyses were performed by MD-L, TD, and LD, with the help of JZ. Data collection was performed by MD-L and LD, with the help of YB. Data interpretation was done by MD-L, LD, TD, and CP, with the help of SB, LH, and SL. Manuscript was written by MD-L and LD, and revised by all authors. CP provided the financial and material resources necessary for the realization of this project.

### Conflict of Interest

The authors declare that the research was conducted in the absence of any commercial or financial relationships that could be construed as a potential conflict of interest.
