# Supplementary material for: Qualitative Changes in Cortical Thymic Epithelial Cells Drive Postpartum Thymic Regeneration
Source: Front Immunol. 2020 Jan 15;10:3118. doi: 10.3389/fimmu.2019.03118 (PMC6974522; doi:10.3389/fimmu.2019.03118)
Supplement: Supplementary file 1 [file Data_Sheet_1.docx]

Supplementary Material

# Supplementary Figures and Tables

## Supplementary Figures


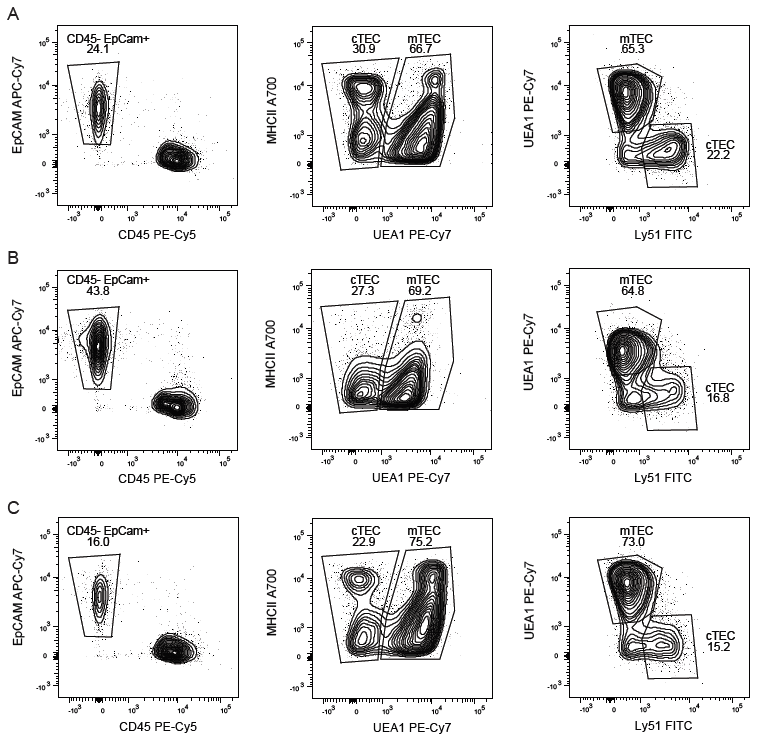
**Supplementary Figure 1.** Representative profile of TECs in (A) NPC, (B) at D-2 and (C) D16 postpartum.


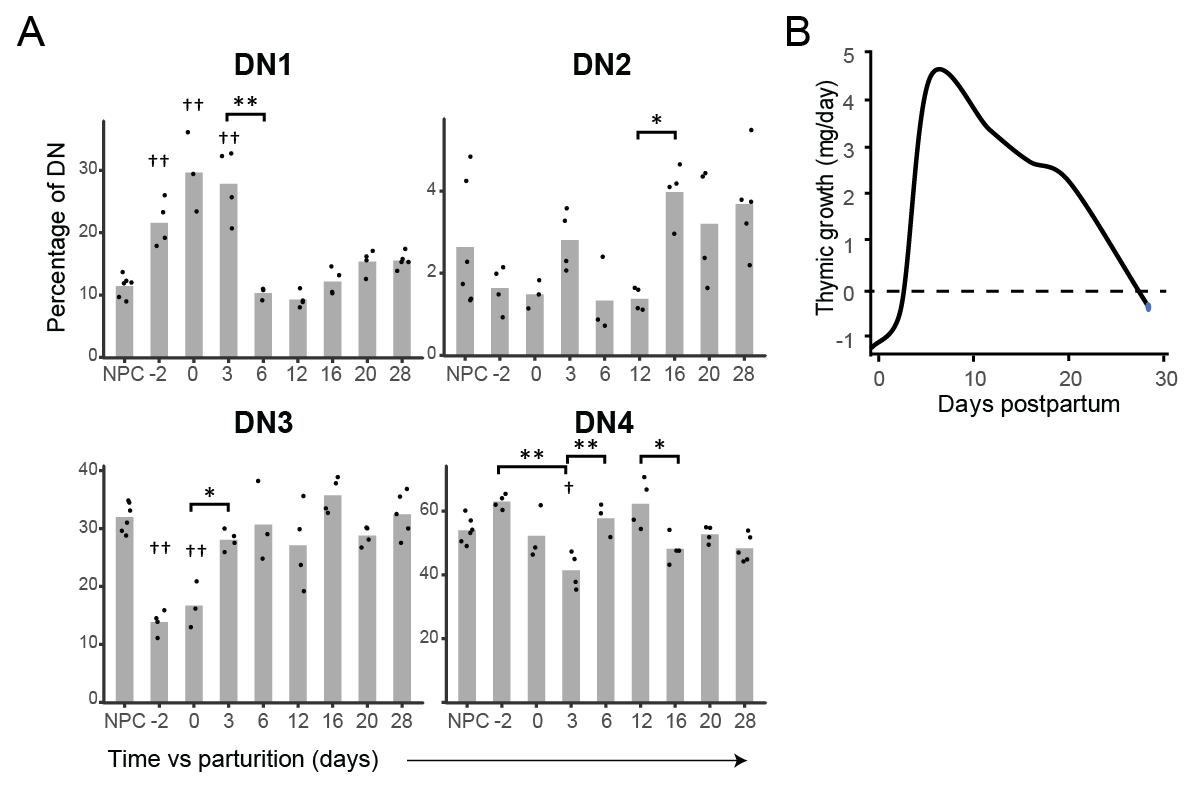


**Supplementary Figure 2.** (A) Percentage of DN1 to DN4 thymocytes over all DN thymocytes (n = 3 to 6). Means and individual replicates are shown as bars and dots, respectively. Significance was assessed with one-way ANOVA followed by post-hoc Tukey test. *p* values are shown against NPC († *p* < 0.05 and †† *p* < 0.01) or against the indicated time point (* *p* < 0.05 and ** *p* < 0.01). (B) Thymic growth rate in late pregnancy and during post-partum regeneration (from E18.5 to D28). The thymic growth (ΔC) was calculated as followed: ∆C= ∆weight/∆time vs previous timepoint.


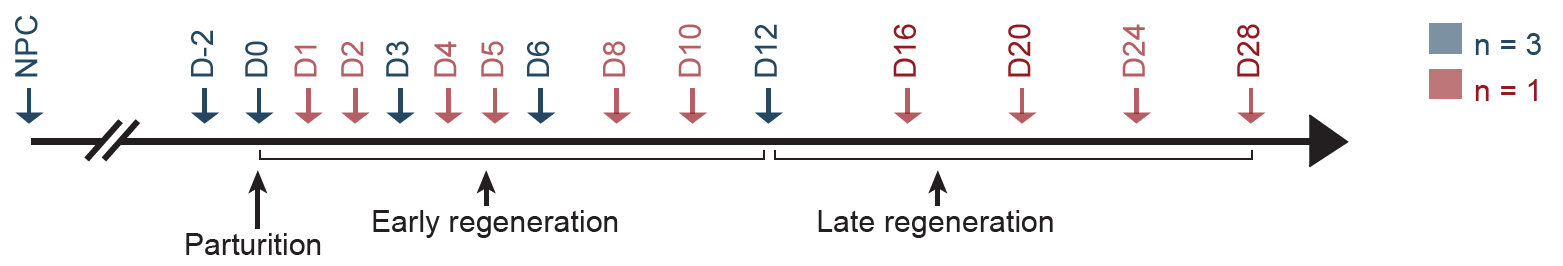


**Supplementary Figure 3.** Schematic representation of RNA sequencing analyses of TECs in late pregnancy and postpartum thymic regeneration. Timepoints for which 3 replicates were analyzed are indicated in blue. We divided the postpartum regeneration period in 2 phases: early regeneration, encompassing all timepoints from D0 to D12, and late regeneration, from D16 to D28.


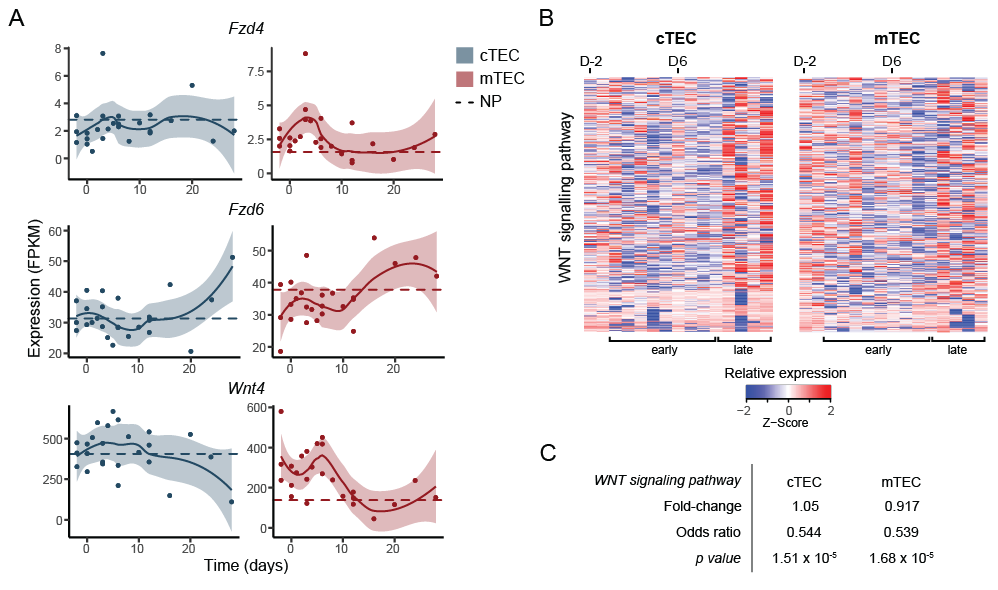


**Supplementary Figure 4**. (A) Expression of WNT receptors *Fzd4* and *Fzd6* and of *Wnt4* in cTECs and mTECs. Each replicate is represented with a dot. Lines represent non-linear regression and the 95% confidence interval are displayed as shaded areas (blue for cTECs, red for mTECs). Average expression in NPC is depicted with a dashed line. (B) Relative expression (Z-score) of genes of the WNT signaling pathway (GO: 0016055). Relative expression (Z-score) from D-2 to D28 is shown. (C) Average fold-change in WNT signaling pathway expression between D-2 and D6. Odds ratio and significance were assessed with Fisher exact test, for fold-change > 2. Gene expression for each time point represents either average expression for triplicates, or raw expression value for single replicate (see Supplementary Figure S2 for number of replicates per timepoint).


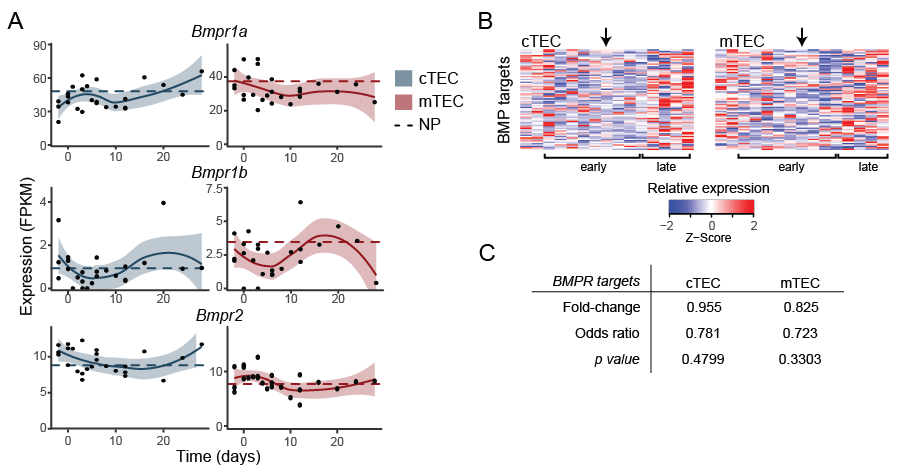


**Supplementary Figure 5**. (A) Expression of BMP4 receptors in cTECs and mTECs. Each replicate is represented with a dot. Lines represent non-linear regression and the 95% confidence interval is displayed as shaded area (blue for cTECs, red for mTECs). Average expression in NPC is depicted with a dashed line. (B) Relative expression (Z-score) of BMPR target genes (GO:0030510). Gene expression is shown as relative expression (Z-score), from D-2 to D28. Arrows indicate D6. (C) Average fold-change in BMPR targets expression between D-2 and D6. Odds ratio and significance were assessed with Fisher exact test, for fold-change > 2. Gene expression for each time point represents either average expression for triplicates, or raw expression value for single replicate (see Supplementary Figure S2 for number of replicates per timepoint).

## Supplementary Tables

Supplementary Table 1. Reagents used for flow cytometry analysis.

| Reagents | Catalog # | Clone | Supplier | Lot # |
| --- | --- | --- | --- | --- |
| Collagenase D | 11088866001 | - | Sigma | 15731423 |
| DNase I | D25-1G | - | Sigma | SLBF7798V |
| Papain | LS003119 | - | Worthington-Biochem | 37J17641 |
| LS Columns | 130-042-401 | - | Miltenyi Biotec | 5180129007 |
| CD326 (EpCAM) Microbeads mouse | 130-105-958 | - | Miltenyi Biotec | 5171027038 |
| EpCAM - APC-Cy7 | 118218 | G8.8 | BioLegend | B244598 |
| CD45 - PE-Cy5 | 553082 | 30-F11 | BD Biosciences | NA |
| UEA1 – biotinylated | B-1065 | - | Vector Laboratories | 0331 |
| I-A/I-E - Alexa Fluor 700 | 107622 | M5/114.15.2 | BioLegend | B217859 |
| I-A/I-E – APC | 107614 | M5/114.15.2 | BioLegend | B191785 |
| Ly51 – FITC | 553160 | 6C3 | Biolegend | 83063 |
| Streptavidin – PE-Cy7 | 557598 | - | BD Biosciences | 5126578 |
| CD4 – Alexa Fluor 700 | 557956 | RM4-5 | BD Biosciences | 7047620 |
| CD4 – BV421 | 100443 | GK1.5 | Biolegend | B223832 |
| CD8a – FITC | 553031 | 53-6.7 | BD Biosciences | 05932 |
| CD11c – PE-Cy7 | 558079 | HL3 | BD Biosciences | 7005642 |
| CD25 – APC-Cy7 | 557658 | PC61 | BD Biosciences | 35781 |
| CD44 – APC | 559250 | IM7 | BD Biosciences | 50592 |
| PE Mouse anti-Human Ki67 set | 556027 | - | BD Biosciences | 5113744 |
| Factor staining buffer set | 00-5523-00 | - | Invitrogen | 1957127 |
| Propidium iodine | 51-66211E | - | BD Biosciences | 6155503 |

**Supplementary Table 2.** Differential expression of genes involved in thymopoiesis in cTECs.

| Genes | Downregulated during pregnancy  (NPC to D-2) | | Upregulated during regeneration  (between D-2 and D3 to D12) | | Correlation with Foxn1 |
| --- | --- | --- | --- | --- | --- |
|  | Fold-change | *p* value | Fold-change | *p* value | R (Pearson) |
| Foxn1 | -1.7x | 0.2336 | 2.0 to 2.7x | p < 0.05 | 1 |
| Dll4 | -5.7x | 0.0009 | 5.3 to 15.0x | p < 0.002 | 0.542 |
| Ccl25 | -4.6x | 0.0456 | 6.6 to 9.3x | p < 0.02 | 0.706 |
| Cxcl12 | -1.8x | 0.0076 | 2.1 to 2.9x | p < 0.008 | 0.462 |
| Il7 | -1.6x | 0.0446 | 1.5 to 1.9x | p < 0.054 | 0.533 |
| Kitl* | -1.1x | n.s. | 2.2x | p < 0.008 | -0.02 |
| Ctsl | -2.3x | 0.0048 | 2.3 to 3.7x | p < 0.003 | 0.133 |
| Prss16 | -7.0x | 0.0058 | 8.3 to 12.9x | p < 0.009 | 0.731 |
| Psmb11 | -6.5x | 0.0450 | 9.0 to 12.8x | p < 0.02 | 0.912 |
| Cd83 | -6.1x | 0.0152 | 5.8 to 12.2x | p < 0.02 | 0.738 |

Significance was assessed using unpaired bilateral student T tests. *For *Kitl*, only the fold-change between D-2 and D6 are shown.
